# Potential drivers of human tick-borne encephalitis in the Örebro region of Sweden, 2010–2021

**DOI:** 10.1038/s41598-023-34675-x

**Published:** 2023-05-11

**Authors:** Lene Jung Kjær, Magnus Johansson, Per-Eric Lindgren, Naveed Asghar, Peter Wilhelmsson, Hans Fredlund, Madeleine Christensson, Amélie Wallenhammar, René Bødker, Gunløg Rasmussen, Petter Kjellander

**Affiliations:** 1 Section for Animal Welfare and Disease Control, Department of Veterinary and Animal Sciences, Faculty of Health and Medical Sciences, University of Copenhagen, Frederiksberg, Denmark; 2 School of Medical Sciences, Faculty of Medicine and Health, Örebro University, Örebro, Sweden; 3 Division of Inflammation and Infection, Department of Biomedical and Clinical Sciences, Linköping University, Linköping, Sweden; 4 Division of Clinical Microbiology, Department of Laboratory Medicine, Region Jönköping County, Jönköping, Sweden; 5 Department of Laboratory Medicine, Faculty of Medicine and Health, Örebro University, Örebro, Sweden; 6 Örebro County Council, Örebro, Sweden; 7 Grimsö Wildlife Research Station, Department of Ecology, Swedish University of Agricultural Sciences (SLU), Riddarhyttan, Sweden

**Keywords:** Diseases, Infectious diseases, Ecological epidemiology

## Abstract

Incidence of tick-borne encephalitis (TBE) has increased during the last years in Scandinavia, but the underlying mechanism is not understood. TBE human case data reported between 2010 and 2021 were aggregated into postal codes within Örebro County, south-central Sweden, along with tick abundance and environmental data to analyse spatial patterns and identify drivers of TBE. We identified a substantial and continuing increase of TBE incidence in Örebro County during the study period. Spatial cluster analyses showed significant hotspots (higher number of cases than expected) in the southern and northern parts of Örebro County, whereas a cold spot (lower number of cases than expected) was found in the central part comprising Örebro municipality. Generalised linear models showed that the risk of acquiring TBE increased by 12.5% and 72.3% for every percent increase in relative humidity and proportion of wetland forest, respectively, whereas the risk decreased by 52.8% for every degree Celsius increase in annual temperature range. However, models had relatively low goodness of fit (R^2^ < 0.27). Results suggest that TBE in Örebro County is spatially clustered, however variables used in this study, i.e., climatic variables, forest cover, water, tick abundance, sheep as indicator species, alone do not explain this pattern.

## Introduction

Tick-borne encephalitis (TBE) is a severe infection that, if symptomatic, can affect the central nervous system and result in lesions in the human brain and spinal cord^1^. TBE is caused by TBE virus (TBEV) belonging to the genus flavivirus of the family Flaviviridae, and consists of three subtypes: European, Siberian, and Far Eastern TBEV^1–3^. TBEV is predominantly tick-borne but has been known to infect via intake of unpasteurised milk products from infected livestock^2–4^. The hard tick *Ixodes persulcatus* is the main vector of the Siberian and Far Eastern subtype, whereas the closely related *Ixodes ricinus* is the main vector for the European TBEV subtype^2^. Morbidity and mortality differ between subtypes, with Far Eastern subtype causing more severe disease and a case-fatality of up to 20–40%^2,3^, compared to milder symptoms and case-fatalities of 6–8% for the Siberian subtype and 1–2% for the European subtype^3^. It has, however, been speculated that this difference in disease severity and case-fatality may at least partly be explained by other factors such as differences in clinical alert and reporting routines which varies between countries^2,5^. As there is no specific antiviral treatment for TBE, active immunisation is the most effective protection against infection with TBEV^4^. In Sweden, recommendations for vaccination vary between geographic areas and counties according to the epidemiological situation, following the recommendations from the Public Health Agency of Sweden^6^ and the county medical officer for communicable diseases.

Incidence of TBE has been increasing globally during the last decades^2,4^ and since 2012, the European Centre for Disease Prevention and Control (ECDC) has included TBE in the list of notifiable diseases in the European Union^7^. The increase in TBE incidence is likely due to several factors such as changes in climate^8,9^ and changes in the availability of tick host species^10,11^, which all impact tick life cycle and thus tick distribution, abundance, and seasonality^11,12^. In Europe, *I. ricinus* has expanded its geographical range northwards and to higher altitudes^9,11,13–15^, whereas *I. persulcatus*, previously limited to eastern Europe and northern Asia, has expanded westward to Finland and northern Sweden^16–18^. In Sweden, *I. ricinu*s is mostly found in the south-central parts of the country^12,19^, whereas *I. persulcatus* thus far has only been found around the northern parts of the Bothnian Bay bordering Finland^16,20^. Consequently, TBEV has also been found at higher altitudes^14,21^ and in areas that were previously non-endemic^8,22^. In Sweden, since 2004, all human TBE cases are notifiable by law, which provides a reliable knowledge of TBE distribution. The endemic area for European TBEV subtype was first described in the Stockholm area^23^ which later expanded to provinces in the south-west and the south of Sweden^13^. TBEV seems to have a focal distributional pattern in endemic areas as the virus is not uniformly present in the tick population^24,25^, and thus the risk for TBE infection is restricted to small geographical foci^26^. During 2021, a record high of 533 TBE cases were reported to the Public Health Agency of Sweden, representing an incidence of 5.1 cases per 100,000 inhabitants, where most of the cases were autochthonous^6^. Although the geographical distribution of TBE cases in Sweden has expanded, most of the cases are reported in southern and central Sweden^6^. In Örebro County, sporadic cases of TBE were reported until 2014 followed by an increase in reported cases over the last years. Since 2016 vaccination is recommended for people at risk of TBEV infection (local county recommendations from the county medical officer for communicable diseases).

The geographical distribution of *Borrelia burgdorferi* and TBEV corresponds to the geographical range of their vectors. However, *B. burgdorferi* demonstrates a fairly even distribution within the range of its vector whereas TBEV shows a patchy distributional pattern, which suggests that other factors may play a part in restricting the range of TBEV^2,27,28^. Studies from arctic Russia and Italy found that the increase in TBE incidence was correlated with changes in (increasing) air temperature^29^, forest structure and roe deer density^30^. In addition, other studies suggest that changes in human activities, such as use of forest resources and changes in agricultural practises, are the ultimate causes of increasing TBE incidence and that these activities are impacted by differences in socio-economic circumstances thus causing different epidemiological patterns in different countries^31,32^.

Several studies have found TBEV-specific antibodies in cattle, goat, and sheep milk^33–35^ and it has been argued that livestock kept on natural pastures and exposed to ticks can be used as sentinels for TBEV distribution and the emergence of new TBEV foci^35^. Wallenhammar et al.^35^ analysed milk and colostrum from sheep and goats and were able to identify three previously unknown TBEV transmission areas within Örebro County, based on TBEV-specific antibodies found within the milk. Thus, analysing milk from pasture-raised livestock may be an interesting and non-invasive surveillance method for revealing presence of TBEV^35^.

In this study we investigated both temporal and spatial patterns as well as potential drivers of TBE using registered human TBE case data from Örebro County in south-central Sweden (59.5350° N and 15.0066° E). We present maps of human TBE incidence and identify areas with high or low clustering of TBE cases within the county. In addition, we explore whether climatic variables, tick abundance, land cover types, and presence of sheep as a sentinel for TBEV incidence and distribution^35^ can explain human TBE spatial patterns and whether handling of sheep could be associated with human TBE cases within the county.

## Methods

### Study area and postal codes

**Figure 1:**
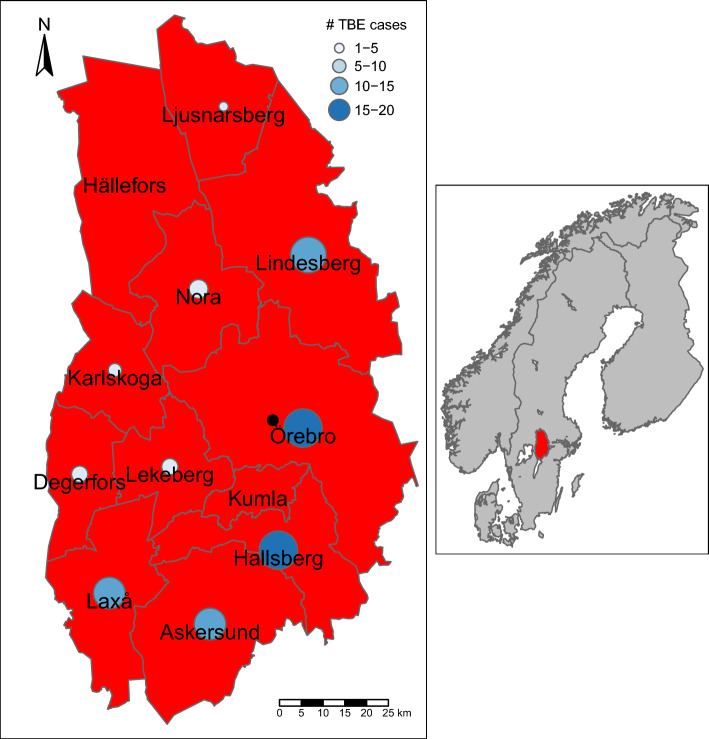
Örebro County with the 12 municipalities depicted. Graduated blue coloured dots depict the number of human TBE cases summarised for 2010–2021 within each postal code. The black dot depicts the county capital of Örebro. Inset map shows the location of Örebro County in Sweden. The map was created using the package tmap^82^ in R 4.1.2^45^.

The study was conducted in Örebro County situated in south-central Sweden (59.5350° N and 15.0066° E), a total area of 8545.6 km^2^. Örebro County is named after its capital city Örebro and consists of 12 municipalities (Fig. 1) accounting for approximately 305,000 residents. Örebro County is surrounded by four major freshwater lakes, having borders with two of them (Vättern and Hjälmaren). The southern part of the county is characterised by agriculture and hemiboreal forests and a climate in-between oceanic and continental with warm summers and cold winters, with mean temperatures below 0 °C during approx. December to February. The northern part of the county is within the boreal zone, agriculture is sparse, and the climate is slightly cooler year-round.

We obtained a shapefile of 291 postal codes in Örebro County from ArcGIS online data^36^, which contained data on area and population size of each postal code.

### TBE data

We obtained data on human TBE cases reported from 2010 to 2021 through the surveillance of the Communicable Diseases Register, called SmiNet, maintained by the Public Health Agency of Sweden^6^. A total of 118 TBE cases were reported in Örebro County within the study period. However, we only included cases where the probable place of exposure, given in the clinical reports, (or patient home address, see below) occurred within Örebro County, i.e., postal codes were within the Örebro region. With these inclusion criteria, 81 cases were included in the study. In 12 of these cases the postal code of the infection site was not known, and thus we used the postal code of these patients’ home address (as all 12 patients resided within Örebro County). We combined the TBE data with the Örebro County postal code shapefile, and based on the postal code population sizes, we calculated the incidence of TBE for each postal code in Örebro County both for individual years and summarised over all 12 years. Furthermore, we calculated the TBE incidence/100,000 inhabitants each year (summarised over all postal codes) using human population data from the shapefile of postal codes in Örebro County from ArcGIS online data^36^. We assumed constant human population size over the 12 years. All methods were performed in accordance with the relevant guidelines and regulations.

### Climate data

We obtained bioclimatic data on annual average temperature, annual temperature range (maximum temperature of warmest month–minimum temperature of coldest month, as a measure of temperature variability), and annual average precipitation from Worldclim^37^. These variables are based on data from 1970 to 2000 and consists of raster images with a resolution of 1 × 1 km. We overlaid this raster with the Örebro postal codes shapefile and aggregated and averaged mean values for each postal code. We furthermore obtained a raster image of annual average relative humidity from the Pale-Blu project^38^ in a resolution of 10 × 10 km. As the relative humidity data was calculated partly based on Worldclim data^37^, this raster data also spanned the years 1970–2000 and as with the bioclimatic data, we aggregated and averaged relative humidity for each postal code.

### Landscape data

As forest type and water may affect tick abundance^39^, we looked specifically at these landscape variables. We obtained land cover data for Örebro County (Nationella Marktäckedata (NMD)^40^) from The Swedish Environmental Protection Agency^41^ as a raster image with a resolution of 10 × 10 m. This raster image contained 25 different land cover classes, ranging from agricultural fields to open water, waterways, and forests. As with the bioclimatic raster images, we overlaid this raster with the Örebro postal codes shapefile and calculated the number of 10 × 10 m water (lakes, rivers, and streams) and forest raster cells within each postal code. To obtain land cover proportions, we multiplied the number of calculated water and forest raster cells by 100 (as each raster cell in the NMD raster represents 100 m^2^) and calculated the respective proportions within each postal code using the postal code area provided by the shapefile. We stratified and merged different forest types within the NMD raster into two forest types: wetland forest and dry forest and also created a stratum called total forest (dry forest + wetland forest, Table 1). Dry forests here represent mainly dry coniferous forest types with a less abundant shrub layer, while wetland forests contain both coniferous and deciduous moist forest types with abundant shrubs layers of bilberry (*Vaccinium myrtillus*), lingonberry (*Vaccinium vitis-idaea*), heather (*Calluna vulgaris*) and mushrooms (Fungi), whereof several species are popular for human consumption (*Cantharellus *sp.* and Boletus *sp., among many other).

**Table 1:** Land cover definition of the Swedish land cover data^40^ and stratification into water, dry- and wetland forest.

Stratification	NMD description
Water	Lakes and streams
Dry forest	Broadleaved forest (not on wetland)
	Mixed coniferous forest (not on wetland)
	Mixed coniferous and deciduous forest (not on wetland)
	Mixed trivial deciduous and broadleaved forest (not on wetland)
	Pine forest (not on wetland)
	Spruce forest (not on wetland)
	Trivial deciduous forest (not on wetland)
Wetland forest	Broadleaved forest (on wetland)
	Mixed coniferous forest (on wetland)
	Mixed coniferous and deciduous forest (on wetland)
	Mixed trivial deciduous and broadleaved forest (on wetland)
	Pine forest (on wetland)
	Spruce forest (on wetland)
	Trivial deciduous forest (on wetland)

### Tick abundance data

As a part of the RåFäst project (“The interplay between ticks, tick-borne diseases and wildlife in Sweden”, the Swedish University of Agricultural Sciences (SLU) at Grimsö Wildlife Research Station^42^), a tick abundance study was conducted in the southern parts of Örebro County. Questing ticks were collected once per site between August 14th and October 16th, 2017, from the hours 10 to 17.30 using the cloth dragging technique^42^. At each site a cloth was dragged 200 m in two directions (north/south and east/west), i.e., 400 m in total, and after every 50 m, the cloths were controlled for ticks. We calculated the mean abundance of tick larvae, nymphs, and adult ticks (both combined as total number of ticks and separately for each life stage and sex) in each postal code encompassed by the RåFäst study by averaging over multiple sites within each postal code. All ticks detected were morphologically identified to species by trained technicians and all were classified as *I. ricinus*. All adult ticks collected were furthermore tested for TBEV, but all ticks tested negative.

### Sheep data

As sheep can be used as a potential sentinel for TBEV incidence^35^, we were interested in investigating the relation between human TBE incidence and the presence and number of sheep farms. We obtained location data on all sheep farms within Sweden, provided by the Swedish Agricultural Agency^43^, and calculated the total number of sheep farms for each postal code within Örebro County.

### Cluster analyses

To explore overall spatial clustering of human TBE cases in Örebro County, we used the spatial autocorrelation tool Global Moran's I in the package sdep^44^ in R 4.1.2^45^. This tool can, in our case, measure spatial autocorrelation based on both postal codes and incidence simultaneously and evaluate whether the county-level observed incidence patterns are significantly clustered, dispersed, or random. We used number of cases summarised over the 12 years and postal code population sizes to calculate the TBE incidence used in Moran’s I. To identify potential local clusters of human TBE cases across postal codes, we used the program SatScan^46^ and the R package rsatscan^47^. We performed spatial scan analyses on the postal code level for summarised years and for separate years with an elliptical scanning window, using the Poisson probability model, where the number of cases within our study area is assumed to be Poisson-distributed, according to a known underlying population at risk (human population within the different postal codes). We used a maximum spatial window size of less than or equal to 50% of the total population at risk. The method identifies significant spatial clusters where there is a higher (hotspots) or lower (cold spots) incidence of cases within the scanning window than expected based on the Poisson probability of the entire study area. SatScan then reports the ratio of observed number of cases within a cluster to the expected number (ODE). Interpretation of an ODE of 1 means that there is no difference from the expected number of cases. We used the Gini coefficient^48^ for cluster selection, as it measures heterogeneity, aiding us in which clusters to report (multiple smaller clusters versus large joint clusters).

### Statistical analysis

To investigate the trend in yearly incidence/100,000 inhabitants between 2010 and 2021, we used R 4.1.2^45^ to fit a logistic and an exponential growth model to our data. Changes in annual incidence are most likely determined by the relationship with one or more external factors (such as tick abundance, available hosts etc.). When such factors are unlimited, incidence rate can be described by an exponential growth model which would be the simplest model to describe an increase in the number of TBE cases directly proportional to incidence at each point in time, with no sign of levelling off. Alternatively, a logistic model could be fitted, implying that incidence is approaching an asymptote as there is some intrinsic regulatory (depressing) response to the number of TBE cases as incidence increase. Here we test for the best fit of either one of these two models to investigate potential trends in the development of future TBE incidence in Örebro County.

To identify potential drivers of TBE occurrence, we ran our statistical analyses as the incidence of TBE cases over the years (2010–2021), calculated as the number of cases out of the total population within a postal code. Due to the limited number of cases within the region, we did not calculate yearly incidence but summarised incidence over all the 12 years. To test for associations between human TBE cases and climatic variables, land cover, tick abundance and sheep farms, we used generalised linear models (GLMs) with a logit link assuming a quasibinomial distribution. A quasibinomial distribution adds an extra parameter (dispersion parameter) compared to the binomial distribution to describe extra variance in the data that cannot be explained by the binomial distribution alone. First, univariable GLMs were used to examine whether there was an association between the dependent and independent variable and thereafter we ran multivariable models with all significant independent variables from the univariable tests. When testing for associations between TBE and tick abundance, only postal codes with estimates of tick abundance were included in the GLMs. For multivariable models, we used backwards stepwise elimination by removing the variable with the highest non-significant P-value and re-running the GLM. We also performed an analysis of variance (ANOVA) between models to check whether a reduction in the residual sum of squares (SS) was statistically significant and compared Quasi AIC-values (QAIC) between models. We checked the final model for spatial autocorrelation by plotting the residuals, calculating Moran’s I and looking at a spline (cross-) correlogram. The latter estimates spatial dependence as a continuous function of distance and shows the correlation of pairs of spatial observations with increasing distance (lag) between them^49^.

### Ethics approval

The data on human TBE cases that were used in our analyses have been collected and stored in the Communicable Disease Register in Örebro County Council and at the Public Health Agency of Sweden. The data used only process TBE cases related to probable place of infection and no personal data. According to the Swedish Act on Ethical Review, there are no requirements to obtain any ethical permissions to use these data sets for analyses as described in this paper.

## Results

### Distribution and incidence of TBE cases within Örebro County

**Figure 2:**
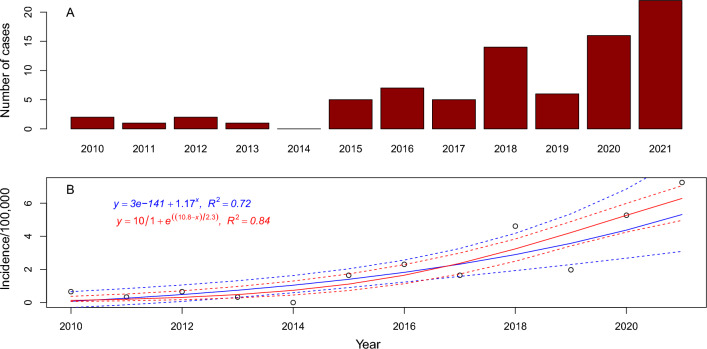
TBE in Örebro County, south-central Sweden from 2010 to 2021. The data are depicted as (**A**) total number of cases (n = 81), and (**B**) incidence and curves fitted from a logistic model (red) and an exponential model (blue) along with their 95% confidence intervals (stippled lines) to show incidence trend based on 12 years. The R^2^ depicted for the logistic model is the Nagelkerke (Cragg and Uhler) pseudo R^2^, calculated using the rcompanion^83^ package in R 4.1.2^45^.

**Figure 3:**
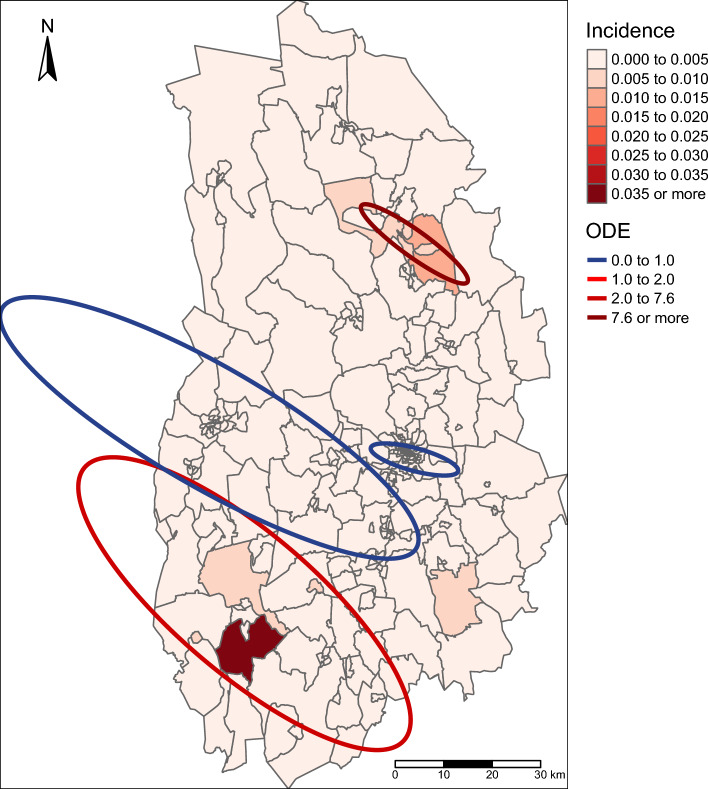
Incidence (depicted at the postal code level) and estimated clusters for the combined years of human TBE (2010–2021). Only significant clusters with the maximum Gini coefficient are depicted. ODE is the observed number of cases divided by expected number of cases, thus red clusters depict hotspots of human TBE cases (more cases than expected), whereas blue clusters depict cold spots (less cases than expected). The map was created using the package tmap^82^ in R 4.1.2^45^.

The 81 human TBE cases between 2010 and 2021 included in this study were distributed over 46 of the 291 different postal codes within Örebro County. The annual incidence of TBE cases in Örebro County showed a substantial increase over the 12-year period, from 0.7 cases/100,000 inhabitants (n = 2) in 2010 to 7.3 cases/100,000 inhabitants (n = 22 cases) in 2021 (assuming constant population size of 303,348 inhabitants obtained from the postal code shapefile from ArcGIS online data^36^) (Fig. 2). The incidence and clustering of TBE cases are depicted at the postal code level in Örebro County in Fig. 3. TBE cases appeared to be located in the south of the county, mainly in postal codes belonging to Laxå municipality and in the north of the county in postal codes belonging to Lindesberg municipality.

### Climate data

Average annual temperature spanned from 6.5 to 7.0 °C in the southern and central parts of Örebro County to 3.5–4.0 °C in the northern parts. Average annual temperature range, which gives a measure of temperature variation over the year, showed a higher variation in the northern part of the county (29–30 °C) compared to the southern part (25–27 °C), with values for the central part ranging in between (Supplementary Fig. S1). Average annual precipitation was generally highest in western postal codes (≥ 700 mm) compared to eastern postal codes (650–750 mm, Supplementary Fig. S1), and average relative humidity was highest in the north of Örebro County, with values ranging from 85.0 to 86.5%. Postal codes in the south and east of the county seemed to have lower relative humidity ranging from 84 to 84.5% (Supplementary Fig. S1).

### Landscape variables

Örebro County consisted of 16.1% forest, which comprised 14.7% dry forest and 1.4% wetland forest, with less forest in postal codes bordering the central city of Örebro. Water in the form of lakes, rivers and streams made up 3.7% of the county. Supplementary Fig. S2 shows the distribution of forest and water proportions across the different postal codes in Örebro County.

### Tick abundance, forest type and sheep farms

In the RåFäst project, 110 sites were sampled within 107 postal codes in southern Örebro County. The total sum of larvae, nymphs and adult ticks was 3144, where the average abundance for all ticks for each postal code spanned from 0 to 200 ticks. The total abundance of each life stage was 1185 larvae (found in 41 postal codes), 1819 nymphs (found in 102 postal codes), 69 adult females (found in 39 postal codes), and 71 adult males (found in 45 postal codes). Among the tick sampling sites, 73 were located in dry forest (mean total tick abundance = 33.95), and 2 in wetland forest (mean total tick abundance = 7). The relative proportions of dry- and wetland forest within the study area were 89% and 11%, respectively. Highest overall abundance was found in postal codes within the municipalities Lekeberg, Laxå, Hallsberg, Kumla, Askersund and Örebro (Supplementary Fig. S3).

The total number of sheep farms in Örebro County was 487 farms within 105 postal codes and the farms were evenly distributed across all postal codes with highest numbers (12–14 farms) in postal codes within Lindesberg and Nora municipalities in the north and Örebro municipality in the central part the county (Supplementary Fig. S4).

### Cluster analyses

**Figure 4:**
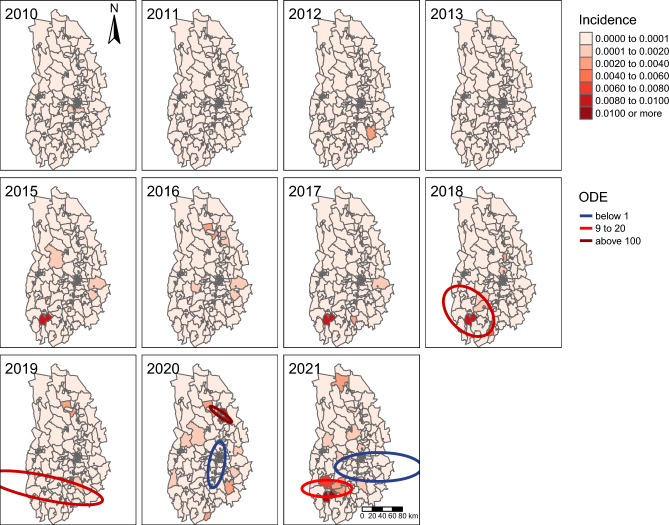
Human TBE incidence (depicted at the postal code level) and estimated clusters for the individual years 2010–2021. Only significant clusters with the maximum Gini coefficient are depicted. ODE is the observed number of cases divided by expected number of cases, thus red clusters depict hotspots of human TBE cases (more cases than expected), whereas blue clusters depict cold spots (less cases than expected). Note that the year 2014 is missing, as no human TBE cases were reported that year. The maps were created using the package tmap^82^ in R 4.1.2^45^.

The Global Moran’s I test to detect overall spatial clustering of human TBE incidence within Örebro County showed that the case data (summarised over all 12 years) were not significantly different from a random distribution (I = 0.034, z = 1.54, p = 0.062), thus indicating that overall county-level TBE incidence was not clustered. For local clustering across postal codes, the SatScan analysis detected significant hotspots for the combined years 2010—2021 in the south-western and north-eastern part of the county, with cold spots in central Örebro County and around Örebro municipality (Fig. 3). For individual years, only the years 2018–2021 had significant clusters with hotspots found in the southern part of the county in 2018, 2019 and 2021 but in the northern part in 2020. A cold spot was found around Örebro municipality in both 2020 and 2021 (Fig. 4).

### Statistical analysis

Fitting both an exponential- and a logistic model to our TBE incidence data, indicated that the logistic model had stronger support compared to the exponential model (R^2^ = 0.84 vs 0.72), implying that incidence will continue to increase but ultimately reach an asymptote according to the logistic model at approximately 10 cases every year/100,000 inhabitants in Örebro County (Fig. 2B). This may however not be accurate as it will also depend on the extent of immunisation within the county.

The dispersion parameters for all GLM models were larger than 0, suggesting that the quasibinomial distribution fitted our data better than a simple binomial distribution (Table 2). The univariable tests showed that incidence of human TBE was significantly associated with mean annual temperature range and mean annual relative humidity, however, no significance was found for average annual temperature and precipitation (Table 2). When separating forest land cover into dry and wetland forest, we found significant associations between both types of forest and incidence of human TBE (Table 2). Combining dry and wetland forest into an all-forest category also showed a significant association with TBE incidence (Table 2). TBE incidence was not significantly associated with tick abundance for any life stage or combined number of ticks. However, we found a significant association between human TBE incidence and the number of sheep farms (Table 2). We suspected that the effect of sheep farms could be related to them being mostly in forested areas, thus we ran a Pearson’s product-moment correlation test between total number of sheep farms and proportion of forest (wetland + dry) within a postal code. The result indicated a significant correlation between these two variables (t_289_ = 8.0, p < 0.001) with a correlation coefficient of 0.43. Thus, number of sheep farms was omitted from further analyses. The multivariable model included the significant variables from the univariable tests, annual mean temperature range, annual mean relative humidity, and dry and wetland forest. In the multivariable model, dry forest was not significant (p = 0.197, Table 3), and removing it from the model produced a lower QAIC (Table 3). Furthermore, an ANOVA comparing the full and reduced model proved no significant differences in the residual SS of the 2 models (Table 3), thus the reduced model including annual mean temperature range, annual mean relative humidity, and wetland forest was chosen as the final model. In the final model, odds ratios showed that for every percent increase in relative humidity and wetland forest, the likelihood of TBE increased by 12.5% and 72.3% respectively (Table 3), whereas for every degree Celsius increase in temperature range, the likelihood of TBE decreased by 52.8%. However, goodness of fit (R^2^) for the final model was less than 0.27, showing poor predictive power.

**Table 2:** GLM results from univariable tests, testing for significant associations between human TBE cases and mean annual temperature (mean temp.), mean annual temperature range (temp. range), mean annual precipitation (mean prec.), mean annual relative humidity (relative humidity), proportion of dry and wetland forest, proportion of water, tick abundance and number of sheep farms.

Variables	Coefficient estimate	t-value	df	p-value	Dispersion parameter
Mean temp.	− 0.646	− 1.289	289	0.198	6.258
Temp. range	− 1.073	− 4.259	289	< 0.0001	4.754
Mean prec.	0.0006	0.962	289	0.337	5.518
Relative humidity	0.132	2.279	289	0.023	5.582
Abundance all ticks	− 0.009	− 0.577	46	0.567	5.008
Abundance larvae	− 0.009	− 0.454	46	0.652	5.274
Abundance nymphs	− 0.020	− 0.620	46	0.539	5.006
Abundance females	0.055	0.105	46	0.916	5.582
Abundance males	0.106	0.201	46	0.841	5.726
Number of sheep farms	0.179	3.044	289	< 0.01	5.883
Dry forest	0.135	2.944	289	< 0.01	4.711
Wetland forest	0.881	5.487	289	< 0.0001	3.232
All forest	0.146	3.472	289	< 0.001	4.138
Water	0.062	1.184	289	0.237	5.733

Note that the sample size and thus degrees of freedom (df) were smaller for models including tick abundance, as we only included postal codes with measures of tick abundance.

**Table 3:** GLM results from the multivariable models, investigating associations between human TBE cases and mean annual temperature range (temp. range), mean annual relative humidity (relative humidity), and the proportion of dry and wetland forest.

Variables	Coefficient estimate	p-value	Anova p-value	OR (CIs)	QAIC
Temp. range	− 0.696	0.001		0.499 (0.327–0.759)	404.350
Relative humidity	0.094	0.102		1.099 (0.982–1.229)	
Dry forest	0.054	0.197		1.056 (0.972–1.146)	
Wetland forest	0.463	0.015		1.589 (1.096–2.302)	
﻿Temp. range	− 0.750	< 0.001	0.195	0.472 (0.309–0.723)	402.300
Relative humidity	0.117	0.029		1.125 (1.012–1.249)	
Wetland forest	0.544	0.003		1.723 (1.205–2.463)	

The ANOVA p-value is from comparing the reduced model to the full model.

*OR* odds ratio, *QAIC* Quasi AIC, *CIs* 95% confidence intervals﻿.

Spatial plots of the residuals of the final model including temperature range, relative humidity and wetland forest showed no particular spatial autocorrelation (Supplementary Fig. S5). Moran’s I also confirmed that the residuals were not significantly different from a random distribution (I = 0.1007, z = 0.28, p = 0.39). However, a spline (cross-) correlogram of the final model residuals showed a generally weak spatial autocorrelation, with weak negative and positive autocorrelation (correlation coefficients between − 0.04 and 0.08) at distances up to 100 km (Supplementary Fig. S6). Despite weak autocorrelation, our results indicate that we did not account for all the spatial variation within the TBE data.

## Discussion

We analysed surveillance data of 81 cases on human TBE with probable place of infection within Örebro County from 2010 to 2021 and identified a ten-fold increase in incidence during this period from 0.7 cases/100,000 inhabitants in 2010 to 7.3 cases/100,000 inhabitants in 2021. Compared to the overall increase in incidence in Sweden from approximately 1.9/100,000 inhabitants in 2010 to 5.1/100,000 inhabitants in 2021, the increase in Örebro County was more drastic than in Sweden as a whole^6^. A similar trend has been reported in Europe where TBE showed a steady increase from 0.6 to 0.9 cases per 100,000 inhabitants from 2016 to 2020^50^. Analysing 12 years of reported TBE incidence in Örebro County, suggested incidence numbers fit a logistic growth curve. If so, incidence in Örebro County seems to be in the rapid growth phase, and we may expect a higher incidence in the future. With the current logistic fit, incidence reaches an asymptote at 10 cases/100,000 inhabitants annually or approximately 30 individual cases per year. However, the fitted model has some uncertainty as seen by the confidence bands in Fig. 2 and in addition assumes that all other factors remain constant (climate, tick abundance, host animals, level of vaccination, human behaviour etc.). This is most likely not the case as factors such as climate, tick abundance, and host animals are highly variable, and furthermore the increasing incidence in Örebro County might give rise to an increase in immunisation and tick protective behaviour within the county. We observed that TBE cases were not evenly distributed within Örebro County, but were spatially clustered across postal codes. The cluster analyses revealed repeated hotspots of TBE in the south of the county and a consistent cold spot with significantly lower incidence around the city of Örebro in the central part of the county. Given the presence of spatial clustering, we sought out to identify potential drivers causing these spatial patterns.

We did not find a significant association between the risk of human TBEV infection and annual mean temperature or precipitation. Temperature, relative humidity, and precipitation has previously been connected to tick activity, development, and abundance^29,51–55^, which may impact TBEV infection rate within ticks and from ticks to hosts. The lack of association for average temperature and precipitation in this study could be due to the climate data being averaged over a long period or the resolution being too coarse to distinguish small variations within a small geographical area such as Örebro County. However, we found a positive association with mean annual relative humidity and a negative association with annual temperature range. Annual temperature range is a measure of variability in temperatures, thus large variability can indicate extreme weather conditions that may affect tick survival, development, and timing of tick activity^56,57^. An in vivo study of *I. ricinus* nymph survival under cold and varying temperatures found that survival rates were lower when exposed to high-frequency temperature variations, suggesting that frequent temperature changes affect tick survival more than low temperatures alone^57^. Furthermore, a study from Great Britain modelling seasonal dynamics of *I. ricinus* found that tick peak emergence times were strongly affected by stochastic temperature variations^58^. These findings could potentially explain why we found significant associations with temperature range and not average annual temperature. The positive association with relative humidity, we found in this study could be explained by ticks being sensitive to desiccation when off-host, and thus restricted to vegetation with a humidity of ≥ 80%^11,59^. Moreover, in vivo studies of TBE infection rates found infection rate to be positively influenced by relative humidity^60,61^, and field studies from southern Norway have found positive associations between relative humidity and TBE incidence in questing *I. ricinus* nymphs^62^, thus relative humidity could have a direct effect on TBEV within the ticks. We observed higher temperature ranges in the north of Örebro County (Supplementary Fig. S1), suggesting that temperature seems to fluctuate more over the year in these areas, which may not be optimal for tick development and activity. However, we also observed high relative humidity in the north of the county and found a small hotspot of TBE incidence towards the north in our cluster analyses. The persistent TBE incidence hotspot in the south-west of Örebro County is in an area with lower temperature ranges and lower relative humidity compared to the northern parts of the county. However, annual means for relative humidity in Örebro County were all above the 80% needed for tick survival. In addition to the effect of climate and weather on ticks and TBEV development and transmission, weather may also affect human behaviour and the frequency of outdoor activities, and thus exposure to potential tick bites and TBEV.

We found that forest was associated with TBE incidence which is in accordance with other studies from Europe. Models on human TBE in Finland and Hungary have both found the amount of forest to be among the top predictors for the distribution of TBE cases^63,64^. These findings seem to be correlated with tick abundance, as the European *I. ricinus* is highly abundant in forested habitats that provide environmental and climatic conditions optimal for tick survival^65–67^. When stratifying forest into dry- and wetland forest, we found that wetland forest had a stronger association with human TBE cases than dry forest. The risk of TBEV infection increased with an increasing proportion of wetland forest even though mean tick abundance was much higher in dry forests compared to wetland forests (though low sample size in wetland forest may attribute to this). Based on previous studies, forest habitats appear to be one of the most favourable for ticks^68^ and high questing activity is most likely related to preserved humidity in such habitats during summer^69^ corresponding to the habitat we classified as wetland forests. However, we cannot rule out that host community composition may differ from dry to wetland forest habitats, explaining a difference in TBE incidence related to unidentified host species. Dry forests offer ideal habitats for many important tick hosts from small rodents, insectivores, and birds to larger mammals such as hares and deer which are believed to have a positive effect on tick abundance^11^. Wetland forests are important habitats for many insectivorous bird species^70^, and birds are known to disseminate TBEV-infected ticks into new areas^71^. In addition, Csank et al.^72^ detected TBEV neutralisation antibodies in a local breeding bird in Drienovská wetland, Slovakia. It has been suggested that birds may play an important role in TBEV ecology as they may act as amplifying hosts or reservoirs^71,72^. This could potentially explain our findings. However, it could also be that our findings may not as much reflect tick abundance but more so human behaviour. In general, wetland forests provide an excellent habitat for berries and mushroom growth^73^, so these habitats may be popular for collecting berries and mushrooms for human consumption. When collecting berries and mushrooms, people tend to wander into the forest off the forest tracks thus exposing themselves to ticks in the understory. In addition, the timing of some berry species (for example bilberry) and mushroom growth and the temporal activity of ticks coincides as ticks are active during fall, which is also when most people collect these berries and mushrooms^74,75^. Unfortunately, we do not have information on the activity performed while contracting TBE (or a tick bite) for any of our human TBE cases, which could have been interesting to include in our models.

With our GLMs, we were not able to establish an association between tick abundance in Örebro County and human TBE cases. It could be due to only a smaller part of the county (approx. 30–40%) being surveyed for tick abundance, and thus we could not run our analysis with all the TBE cases in the region. It could, however, also be due to other factors affecting the distribution of TBE cases, not considered in our model, such as availability of host species or forest structure^73^. Sheep are considered important tick hosts where they occur, and it has previously been demonstrated that sheep may function as a sentinel species for TBEV incidence^35^. Furthermore, humans herding and working with sheep are most likely more outdoors in habitats harbouring ticks, which may make them more susceptible to tick bites and potentially TBEV. Thus, we here tested for associations between human TBE incidence and the number of sheep farms in Örebro County. We did find a significant association; however, sheep farms were strongly related to forest habitats, making it impossible to separate the cause and effect between the two. Sheep farms were thus omitted as a predictor in our model.

Other studies have found that host species composition and abundance^30,64^ are among the drivers of TBEV, thus incorporating host data could potentially improve our models. However, no fine-scale data on host composition and density are available for Örebro County. Larger host species in Sweden are only monitored through the hunting bags, although moose density is estimated using pellet counts. Hunting bag statistics in Örebro County is reported from 10 different hunting district distributed throughout the county, where on average approx. 0.3 roe deer, 0.3 fallow deer, 0.1 red deer and 0.5 wild boar/km^2^ are reported killed each year, with pronounced differences between districts^76^. Moose density in Örebro County is estimated to be less than 0.5/km^2^^76^. Generally, the hunting bag in Örebro County is higher in southern and eastern districts, and whereas the total yearly hunting bag is not very different from neighbouring counties, it is much lower compared to southern Sweden^76^. Örebro County is surrounded by lakes and bordering the two large lakes Vättern and Hjälmaren, that attract migrating birds during the spring and autumn migrations^77^. These birds may introduce TBEV-infested ticks to the county and could thus be of importance when investigating spatial patterns of TBE in Örebro. Unfortunately, we do not have data on migrating birds for the entire Örebro region, but this may be considered for future studies. The establishment of TBEV within a region is furthermore dependent on the microclimate and population density of small rodent hosts^13^. Warmer climate may be a positive factor for both ticks and hosts and increases the possibility for TBEV establishment due to viral transmission via co-feeding of larvae and nymphs^78–80^. Although we found associations of human TBE incidence and climate in our study, the climate data was of low resolution, and microclimatic data may be more informative when studying TBEV and possible host species. Unfortunately, no data on micro climatic conditions exists for all postal codes.

During the study period we identified TBE hotspots in the southern and northern parts of the county. This trend indicates that TBEV has the potential to establish permanently in the region. Other studies have also found that TBEV may remain endemic in the same area over many years—for example genetic analyses revealed establishment of specific TBEV strains in certain regions, which persisted even after decades^81,82^. However, we found no evidence of a higher amount of wetland forest habitats within the postal codes in the clusters. A significant incidence cold spot around Örebro City is not surprising as this area has very little forest and highly urbanised surroundings.

We aggregated our data to the postal code level using postal codes of probable infection site. Furthermore, we used the home address postal code of 12 TBE cases, as information on infection sites were lacking. Both the latter and the coarse aggregation of our data can obfuscate details that may have importance in the occurrence of TBEV. A possible solution could have been to assume that most infections happen close to home and only used data on patient home addresses. However, a given probable place of infection seemed more accurate. Moreover, due to guidelines on human health research ethics, we were not allowed to get exact addresses on human TBE patients, thus limiting our study to aggregation by postal codes.

## Conclusion

We found that human TBE incidence in Örebro County has been increasing continuously since 2010 resembling a logistic growth curve and thus may potentially stabilize in the future. TBE cases were spatially clustered, with higher incidence in the south and a consistently low incidence in postal codes in and around Örebro City. The TBE incidence was associated with temperature variability, relative humidity, and the proportion of forest within a postal code, particularly the amount of wetland forest. However, our models had relatively low goodness of fit, suggesting that other factors not considered in this study may be important for the spatial distribution of TBE within Örebro County. The explored drivers of TBE (climate and land cover) were not changing over time and the underlying causes of the geographical spread of human TBE cases to Örebro County and the ongoing increase in human incidence remain unexplained.

## Supplementary Information

Supplementary Figures.

## Data Availability

The data on human TBE cases that were used in our analyses have been collected and stored in the Communicable Disease Register in Örebro County Council and at the Public Health Agency of Sweden. Maps of Region Örebro County with human TBE cases plotted according to presumed place of infection are available at https://vardgivare.regionorebrolan.se/sv/vardriktlinjer-och-kunskapsstod/smittskydd/vaccinationer/tbe-vaccin/. Furthermore, TBE county level incidence data are available at https://www.folkhalsomyndigheten.se/folkhalsorapportering-statistik/statistik-a-o/sjukdomsstatistik/tick-borne-encephalitis-tbe/?t=county.
