# Supplementary material for: Potential drivers of human tick-borne encephalitis in the Örebro region of Sweden, 2010–2021
Source: Sci Rep. 2023 May 11;13:7685. doi: 10.1038/s41598-023-34675-x (PMC10175290; doi:10.1038/s41598-023-34675-x)
Supplement: Supplementary file 1 — Supplementary Figures. [file 41598_2023_34675_MOESM1_ESM.pdf]

**Supplementary material for:**

# Potential drivers of human tick-borne encephalitis in the Örebro region of Sweden, 2010-2021

Lene Jung Kjær[1*], Magnus Johansson[2], Per-Eric Lindgren[3,4], Naveed Asghar[2], Peter Wilhelmsson[3,4], Hans Fredlund[5,6], Madeleine Christensson[7], Amélie Wallenhammar[2], René Bødker[1], Gunløg Rasmussen[2,6,†], and Petter Kjellander[7,†]

[1]Section for Animal Welfare and Disease Control, Department of Veterinary and Animal Sciences, Faculty of Health and Medical Sciences, University of Copenhagen, Frederiksberg, Denmark
[2]School of Medical Sciences, Faculty of Medicine and Health, Örebro University, Örebro, Sweden
[3]Department of Biomedical and Clinical Sciences, Division of Inflammation and Infection, Linköping University, Linköping, Sweden
[4]Division of Clinical Microbiology, Department of Laboratory Medicine, Region Jönköping County, Jönköping, Sweden
[5]Department of Laboratory Medicine, Faculty of Medicine and Health, Örebro University, Örebro, Sweden
[6]Örebro County Council, Örebro, Sweden
[7]Grimsö Wildlife Research Station, Department of Ecology, Swedish university of Agricultural Sciences (SLU), Riddarhyttan, Sweden

*Corresponding author:
Email: lenju@sund.ku.dk

[†]Authors contributed equally.

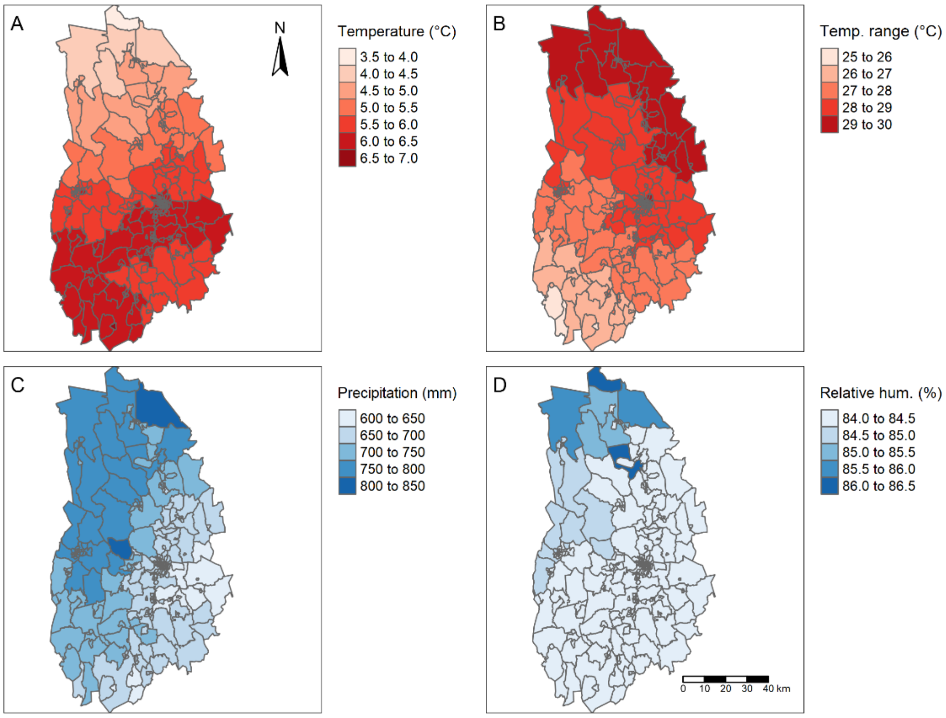


**Figure S1.** Annual means of A) temperature, B) temperature range (maximum temperature of warmest month - minimum temperature of coldest month), C) precipitation and C) relative humidity in the different postal codes within Örebro County, Sweden. The maps were created using the package tmap[1] in R 4.1.2[2].

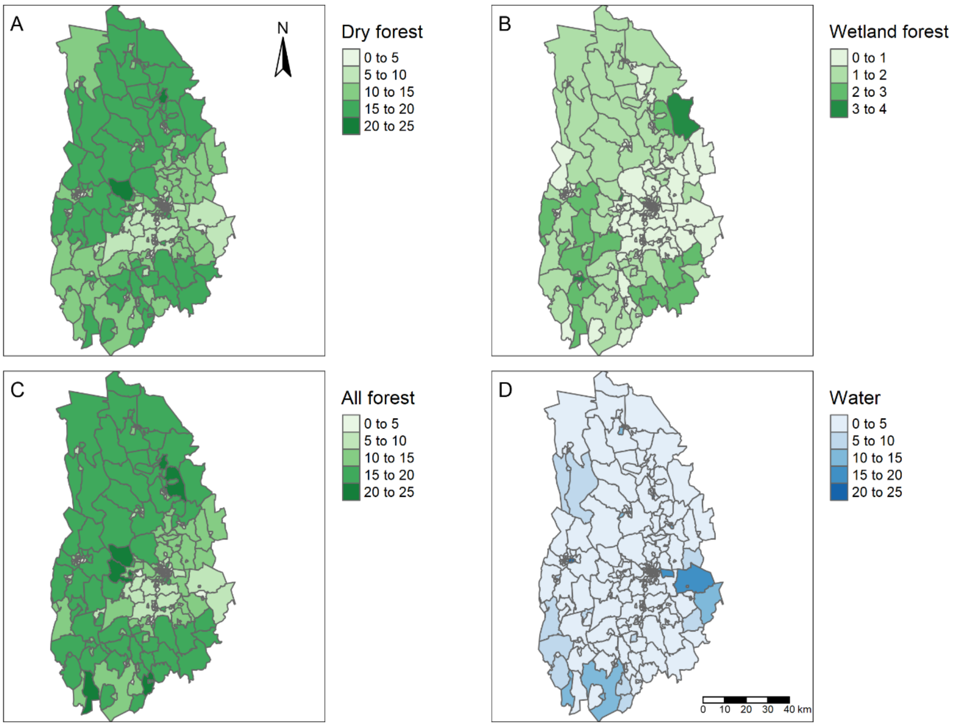


**Figure S2.** The proportion of A) dry forest, B) wetland forest, C) all forest (wetland + dry forest) and C) water (lakes, rivers and streams) in the different postal codes within Örebro County, Sweden. Note that the scale is much smaller for wetland forest. The maps were created using the package tmap[1] in R 4.1.2[2].

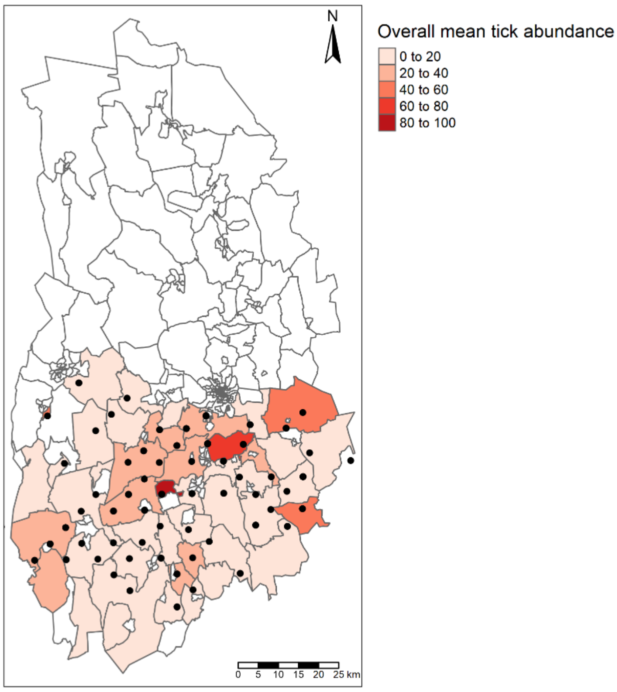


**Figure S3.** Mean abundance of ticks (larvae, nymphs and adult *I. ricinus*) within each Örebro County postal code averaged over multiple study sites (black dots) from the RåFäst project. The eastern-most study site was not within the borders of our postal code shape file for Örebro County and was omitted from our analyses. White areas are postal codes not sampled for tick abundance. The map was created using the package tmap[1] in R 4.1.2[2].

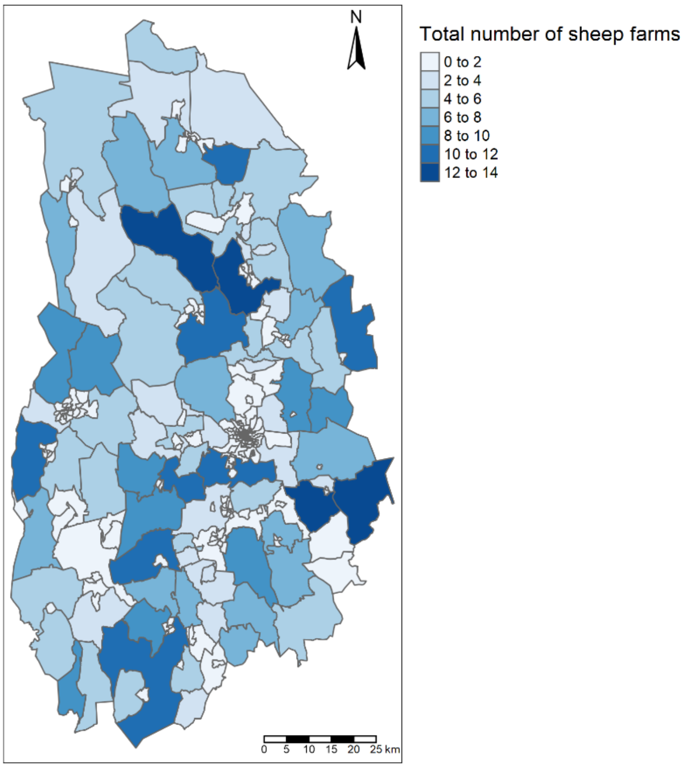


**Figure S4.** Total number of sheep farms within each postal code in Örebro County, Sweden. The map was created using the package tmap[1] in R 4.1.2[2].

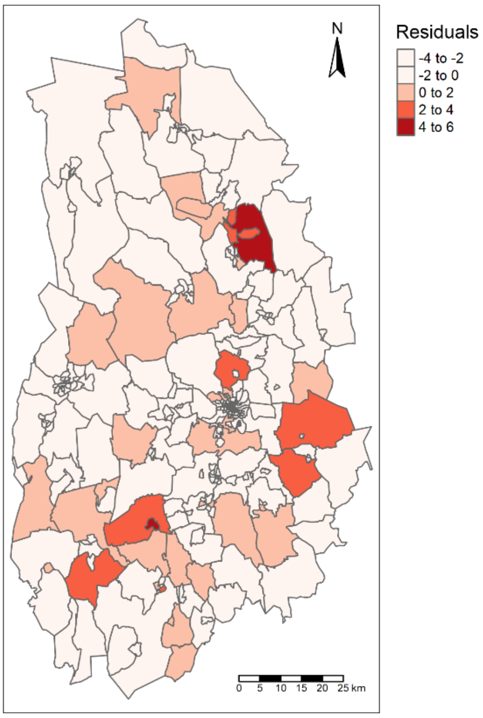


**Figure S5.** Residual plot of the final TBE model including mean annual temperature range, mean annual relative humidity, and wetland forest. The map was created using the package tmap[1] in R 4.1.2[2].

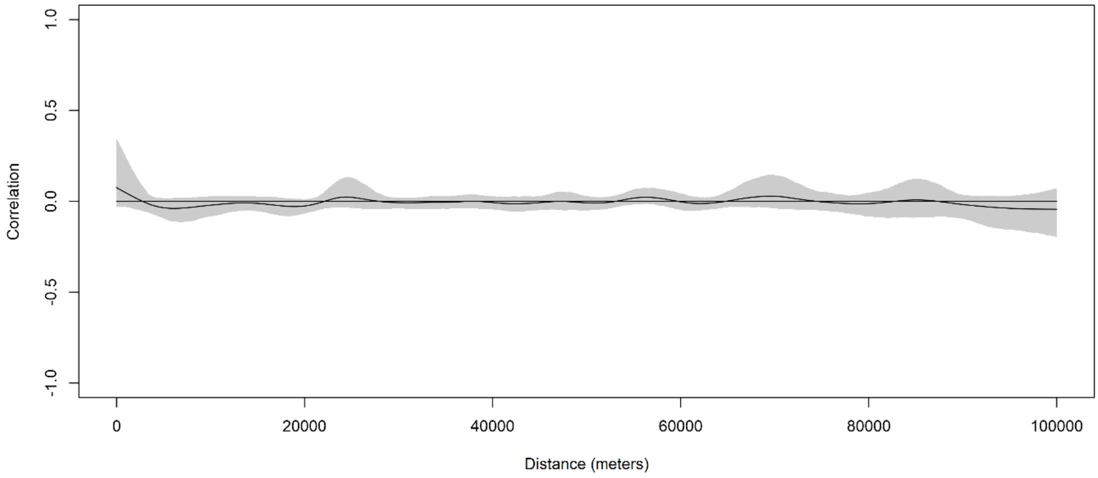


**Figure S6.** Spline (cross-) correlogram of the final TBE model residuals, showing the spatial autocorrelation between residuals and their bootstrapped 95% confidence intervals at distances up to 100 kilometres. The Spline correlogram was calculated using centroid coordinates of each postal code region and the ncf package[3] in R 4.1.2[2]

## References

1. Tennekes, M. tmap: Thematic Maps in R. *J. Stat. Softw.* **84**, 1-39 (2018).

2. R Development Core Team. R: A Language and Environment for Statistical Computing. R Foundation for Statistical Computing http://www.r-project.org (2022).

3. Bjørnstad, O. N. & Falck, W. Nonparametric spatial covariance functions: Estimation and testing. *Environ. Ecol. Stat.* **8**, 53-70 (2001).
